# Continuity of GP care is associated with lower use of complementary and alternative medical providers: a population-based cross-sectional survey

**DOI:** 10.1186/s12913-014-0629-7

**Published:** 2014-12-10

**Authors:** Anne Helen Hansen, Agnete E Kristoffersen, Olaug S Lian, Peder A Halvorsen

**Affiliations:** Norwegian Centre for Integrated Care and Telemedicine, University Hospital of North Norway, PO Box 35, 9038 Tromsø, Norway; The National Research Center in Complementary and Alternative Medicine (NAFKAM), University of Tromsø - The Arctic University of Norway, Tromsø, Norway; Faculty of Health Sciences, Department of Community Medicine, University of Tromsø - The Arctic University of Norway, Tromsø, Norway; General Practice Research Unit, Department of Community Medicine, University of Tromsø - The Arctic University of Norway, Tromsø, Norway

**Keywords:** Continuity of patient care, General practice, Primary health care, Complementary and alternative medical providers, Cross-sectional study, Norway

## Abstract

**Background:**

Continuity of general practitioner (GP) care is associated with reduced use of emergency departments, hospitalisation, and outpatient specialist services. Evidence about the relationship between continuity and use of complementary and alternative medical (CAM) providers has so far been lacking. The aim of this study was to test the association between continuity of GP care and the use of CAM providers.

**Methods:**

We used questionnaire data from the sixth Tromsø Study, conducted in 2007–8. Using descriptive statistical methods, we estimated the proportion using a CAM provider among adults (30–87 years) who had visited a GP during the last 12 months. By means of logistic regressions, we studied the association between the duration of the GP-patient relationship and the use of CAM providers. Analyses were adjusted for the frequency of GP visits, gender, age, marital status, income, education, and self-rated health and other proxies for health care needs.

**Results:**

Of 9,743 eligible GP users, 85.1% had seen the same GP for more than two years, 83.7% among women and 86.9% among men. The probability of visiting a CAM provider was lower among those with a GP relationship of more than 2 years compared to those with a shorter GP relationship (odds ratio [OR] 0.81, 95% confidence interval [CI] 0.68-0.96). Other factors associated with CAM use were female gender, poor health, low age and high income. There was no association with education.

**Conclusions:**

Continuity of GP care as measured by the duration of the GP-patient relationship was associated with lower use of CAM providers. Together with previous studies this suggests that continuity of GP care may contribute to health care delivery from fewer providers.

## Background

Continuity of general practitioner (GP) care is commonly defined as a relationship between a single practitioner and a patient that extends beyond specific episodes of illness or disease [1]. Continuity is assumed to be associated with quality and efficiency in delivering health care, and therefore of great value [2]. This paper is concerned with personal or relational continuity given by one practitioner over a defined time. Such longitudinal care is often measured as the duration of the patient-doctor relationship [3].

Continuity of GP care is highly valued by patients [4], and is believed to have few negative consequences [5,6]. It is suggested to increase patient compliance [7], patient and doctor satisfaction [3,5], and comprehensiveness of care [8], and to enhance receipt of preventive services, to decrease duplication of services and the use of emergency departments [9], hospitalisation, and outpatient specialist services [10]. Continuity of GP care is threatened by changes in society and health services, and this trend is likely to continue in the future [10-13].

Definitions of complementary and alternative medical (CAM) providers vary between countries and organisations. Here, we defined CAM providers as “providers others than authorised health personnel who give health-related treatment outside the established health services.” This definition conforms with the Norwegian law on alternative treatment [14]. Chiropractors are authorised health personnel in Norway [15], and so are not included as CAM providers in this study.

The use of CAM providers has increased in Europe in recent years [16]. Use in Norway is higher among women than men, and higher among younger and middle aged people [17]. Patients visit CAM providers due to negative communication experiences with doctors [18], distrust in traditional health care [19], trust in CAM providers [20], and a desire to achieve a more holistic view, active participation, and empowerment in their care [20,21]. Most patients do not discuss their CAM treatment with their GP [22]. However, some treatment by CAM providers can interact with GP treatment in ways that may or may not be beneficial to the patient [23].

Tromsø is the largest city in North Norway with around 72,000 inhabitants and 64 GPs (38% women) [24]. On the basis of voluntary registration in The Register of CAM Practitioners by 35 providers [25] and personal observations (unpublished observations by AEK), we estimate the number of CAM providers in Tromsø to be around 50.

The Norwegian patient list system was implemented in 2001, with the aim of improving quality, accessibility, and continuity in general practice by providing all residents with a regular doctor. Tromsø municipality has run the patient list system with personal lists since 1993, initially as a pilot scheme. The average list size is 1,230 [24]. Practices consist of 4–6 GPs with personal lists. GPs are well regarded [26], and only 0.4% of the population has chosen to remain outside GPs’ lists [27]. Together with universal insurance and gatekeeping, the list system provides strong incentives for personal continuity of care, and 92% of the population report that they have a current GP that they usually consult [28]. Residents can change GP twice a year without providing reasons, and about 44% of the GP lists were open for new patients in 2008 [29]. About half of the doctor changes in Norway occur because the doctor moves or discontinues the practice [27]. Adults make a small co-payment for GP visits, whereas visits to CAM providers are fully paid by the users. The GP’s gatekeeper role does not apply to the use of CAM providers.

GP and CAM services are linked by the fact that 8.4% of the population seek health care from both during a year [30], and the use of CAM has been described as a public health issue [31]. In the light of the reasons stated for seeking care from CAM providers [18-21], it seems feasible that patients with continuity might obtain more of these qualities from their GP, and thus be less likely to visit CAM providers. Similarly, one might expect continuity of care to be associated with lower use of CAM providers because it is associated with lower use of other health services [9,10]. An understanding of whether longitudinal continuity of GP care is associated with lower use of CAM providers is relevant because it may influence GPs’ and CAM providers’ awareness of each other, with possible consequences for communication, cooperation, and clinical practice. In addition, enhanced knowledge in this area may have significance for planning and organising health services. However, evidence about whether continuity of GP care may be associated with the use of CAM providers has been lacking. Our research question was therefore articulated as follows: How is longitudinal continuity of GP care associated with the use of CAM providers in an adult population?

Our aim in the present study was to investigate this by testing whether self-reported use of CAM providers was associated with self-reported duration of the GP-patient relationship. We hypothesised that a longer duration of the GP-patient relationship would be associated with a reduced likelihood of using CAM providers.

## Methods

### Data

For this cross-sectional study we used survey data from the sixth Tromsø Study (Tromsø 6), conducted from October 2007 to December 2008. The survey consisted of questionnaires, clinical examination and laboratory tests. Four groups were invited: every resident aged 40–42 or 60–87 years (n = 12,578), a 10% random sample of individuals aged 30–39 (n = 1,056), a 40% random sample of people aged 43–59 (n = 5,787) and all subjects who had attended the second visit of the fourth Tromsø Study, if not already included in the other three groups (n = 341). The sampling reflected the need for repeated measurements and follow-up as well as the need to enrol new participants for ongoing and new projects.

Our data were retrieved from the two self-administered questionnaires. The first was mailed with the invitation about two weeks ahead of the suggested appointment time. Participants were invited to attend whenever suitable within the survey opening hours (between 09:00 and 18:00). Non-respondents were given one reminder. Those who attended received an explanatory statement and gave their informed consent. The second questionnaire was handed out, and most participants completed it while waiting for the clinical examination. The comprehensive Tromsø 6 data include self-reported demographic and socio-economic characteristics, and information about symptoms and diseases, health status, and use of medicines and health care services. Both questionnaires and further details about enrolment methods, attendees and non-attendees are available in English at the Tromsø Study website [32] and elsewhere [33]. The sixth Tromsø Study has been approved by the Regional Committee for Medical and Health Research Ethics (REK 2009/2536).

Self-reported survey data are probably the best source of data for studies of CAM provider use in Norway since CAM providers in general are not required to keep records, and registry data is lacking.

### Participants

**Figure 1:**
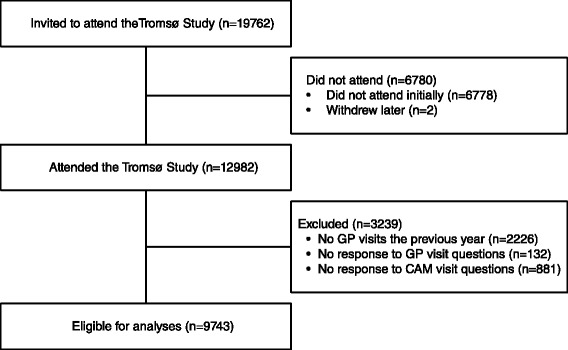
**Flow chart of study population.**

To ensure that there was an ongoing therapeutic relationship with the GP, we excluded participants who reported no GP visits during the previous 12 months (n = 2,226). We also excluded those who failed to answer the questions about use of GP (n = 132) or CAM providers (n = 881). The final sample consisted of 9,743 participants (Figure 1). For 734 participants (7.5%) who reported use of a GP but not the number of visits, we substituted missing values with the average number of visits (given at least one) within each gender and 10-year age group.

### Variables

Participants were asked if they had visited various health care services, including GPs and CAM providers, during the previous year; and if so, how many times. The dependent dichotomous variable was use of CAM providers at least once during the previous year, obtained from the question “Have you during the last 12 months visited an alternative practitioner (homeopath, acupuncturist, foot zone therapist, herbal medicine practitioner, laying on hands practitioner, healer, clairvoyant etc.)?” [32].

The key independent variable for measuring continuity of care was the duration of the GP-patient relationship (GP duration), obtained from the question “For how long have you had your current GP/other doctor?” The response options were dichotomised into two years or less and more than two years (the longest response alternative).

The adjustment independent variables were frequency of GP visits in the previous year, gender, age, marital status, income, education, and self-rated health. Intensity of GP care was measured by the variable frequency of GP visits during the previous year (GP frequency). Responses were dichotomised by median split, and those with 3 or more GP visits were grouped as frequent users. Age was grouped in 20-year age groups. For marital status we used the original response options: married/cohabitant or single. The income variable referred to the household’s total gross income in the year prior to the study. Eight original response categories were merged into low income (< NOK 200,000), low middle income (NOK 201,000-400,000), high middle income (NOK 401,000-700,000) and high income (> NOK 700,000). We defined three education response categories from the original five: low (primary and part of secondary school), middle (high school) and high education (college or university). Response options for the self-rated health variable were reduced from five original categories (very bad - bad - fair - good - excellent) to four by merging the bad and very bad categories, due to the low numbers that they contained.

### Analyses

Data were analysed by means of descriptive statistics and logistic regressions. Correlations were tested with Pearson’s and Spearman’s correlation coefficients. We made a univariable logistic regression with the dependent variable and the key independent variable of GP duration. The independent variables in the multivariable regression (GP duration, GP frequency, gender, age, marital status, income, education, and self-rated health) were introduced collectively into the model.

For validation purposes we performed multivariable regressions where the variable self-rated health was substituted with other need equivalents (psychological problems for which help had been sought, persistent or constantly recurring pain that had lasted for 3 months or more, persistent musculoskeletal pain for at least 3 of the last 12 months, and the EQ-5D score scale [34]). First-order interactions were tested by introducing interaction terms in the regression models.

We used 95% confidence intervals (CI) throughout the study. All analyses were accomplished using Stata, version 12.0.

## Results

In total, 12,982 persons aged 30–87 years participated in Tromsø 6, constituting an overall response rate of 65.7% [32,33] (Figure 1). The participants comprised 33.8% of the total population in that age group within Tromsø municipality.

Among those who had visited a GP during the previous year, 13.3% had also visited a CAM provider: 17.2% of the women and 8.7% of the men (Table 1). Frequent GP visitors had higher CAM visit rates (Table 1). Among those with GP duration of more than 2 years, 12.5% had visited a CAM provider, whereas 17.2% with a shorter GP duration had visited a CAM provider in the previous year (Table 1). The mean age of GP visitors and CAM visitors was 57.9 (57.6-58.1) and 55.7 (55.0-56.3) years, respectively. Of the GP users, 54.9% were female and 62.8% had good/excellent self-rated health (Table 2). Of the CAM visitors, 70.7% were female and 54.0% had good/excellent self-rated health (Table 2).

**Table 1:** **Proportion visiting CAM providers at least once during the previous year**

	**Both genders**		**Women**		**Men**	
	**n/N**	**%**	**n/N**	**%**	**n/N**	**%**
**Total sample**	1300/9743	13.3	919/5346	17.2	381/4397	8.7
**GP duration**						
0-2 years	230/1339	17.2	175/804	21.8	55/535	10.3
>2 years	961/7671	12.5	668/4117	16.2	293/3554	8.2
**GP frequency**						
< 3 visits	489/4875	10.0	305/2445	12.5	184/2430	7.6
3 visits +	811/4868	16.7	614/2901	21.2	197/1967	10.0
**Age**						
30-49	466/2935	15.9	339/1704	19.9	127/1231	10.3
50-69	652/4948	13.2	449/2627	17.1	203/2321	8.8
70-87	182/1860	9.8	131/1015	12.9	51/845	6.0
**Marital status**						
Single	367/2377	15.4	295/1618	18.2	72/759	9.5
Married/cohabitant	890/7105	12.5	588/3534	16.6	302/3571	8.5
Household income*						
Low	134/1107	12.1	114/763	14.9	20/344	5.8
Low middle	354/2529	14.0	250/1439	17.4	104/1090	9.5
High middle	438/3200	13.7	292/1555	18.8	146/1645	8.9
High	276/2196	12.6	182/1066	17.1	94/1130	8.3
Education**						
Low	349/2801	12.5	252/1684	15.0	97/1117	8.7
Middle	437/3291	13.3	299/1714	17.4	138/1577	8.8
High	503/3535	14.2	360/1882	19.1	143/1653	8.7
**Self-rated health**						
Poor	142/596	23.8	100/348	28.7	42/248	16.9
Fair	451/2996	15.1	320/1636	19.6	131/1360	9.6
Good	575/4871	11.8	404/2617	15.4	171/2254	7.6
Excellent	119/1211	9.8	86/710	12.3	33/510	6.5

*CAM* complementary and alternative medical provider; *GP* general practitioner.

*Low (< NOK 200,000), Low middle (NOK 201,000-400,000), High middle (NOK 401,000-700,000), High (> NOK 700,000).

**Low (primary/part of secondary school), Middle (high school), High (college/university).

**Table 2:** **Characteristics of GP users and CAM users (%)**

	GP users*			GP + CAM users**		
	**Both genders**	**Women (54.9** **%)**	**Men (45.1** **%)**	**Both genders**	**Women (70.7** **%)**	**Men (29.3** **%)**
**GP duration**	n = 9010	n = 4921	n = 4089	n = 1191	n = 843	n = 348
0-2 years	14.9	16.3	13.1	19.3	20.8	15.8
>2 years	85.1	83.7	86.9	80.7	79.2	84.2
**GP frequency**	n = 9743	n = 5346	n = 4397	n = 1300	n = 919	n = 381
< 3 visits	50.0	45.7	55.3	37.6	33.2	48.3
3 visits +	50.0	54.3	44.7	62.4	66.8	51.7
**Age**	n = 9743	n = 5346	n = 4397	n = 1300	n = 919	n = 381
30-49	30.1	31.9	28.0	35.9	36.9	33.3
50-69	50.8	49.1	52.8	50.1	48.9	53.3
70-87	19.1	19.0	19.2	14.0	14.2	13.4
**Marital status**	n = 9482	n = 5152	n = 4330	n = 1257	n = 883	n = 374
Single	25.1	31.4	17.5	29.2	33.4	19.3
Married/cohabitant	74.9	68.6	82.5	70.8	66.6	80.7
Household income***	n = 9032	n = 4823	n = 4209	n = 1202	n = 838	n = 364
Low	12.3	15.8	8.2	11.2	13.6	5.5
Low middle	28.0	29.8	25.9	29.4	29.8	28.6
High middle	35.4	32.3	39.1	36.4	34.9	40.1
High	24.3	22.1	26.8	23.0	21.7	25.8
Education****	n = 9627	n = 5280	n = 4347	n = 1289	n = 911	n = 378
Low	29.1	31.9	25.7	27.1	27.7	25.7
Middle	34.2	32.5	36.3	33.9	32.8	36.5
High	36.7	35.6	38.0	39.0	39.5	37.8
**Self-rated health**	n = 9674	n = 5302	n = 4372	n = 1287	n = 910	n = 377
Poor	6.2	6.6	5.7	11.0	11.0	11.1
Fair	31.0	30.8	31.1	35.0	35.2	34.8
Good	50.3	49.4	51.5	44.7	44.4	45.4
Excellent	12.5	13.2	11.7	9.3	9.4	8.7

*GP* general practitioner; *CAM* complementary and alternative medical provider.

*One or more GP visits the previous 12 months.

**GP users with one or more CAM visits the previous 12 months.

***Low (< NOK 200,000), Low middle (NOK 201,000-400,000), High middle (NOK 401,000-700,000), High (> NOK 700,000).

****Low (primary/part of secondary school), Middle (high school), High (college/university).

The duration of the GP-patient relationship was more than two years for 85.1% of the sample: 83.7% among women and 86.9% among men (Table 2). Among those who rated their health as bad/fair and good/excellent, GP duration was more than two years for 84.4% and 85.3%, respectively.

In univariable logistic regression analysis, the probability of visiting a CAM provider was lower among those with a long GP-patient relationship (OR 0.69, CI 0.59-0.81). The association was sustained after adjustment for GP frequency, gender, age, marital status, income, education, and self-rated health (OR 0.81, CI 0.68-0.96) (Table 3). The overall association remained in multivariable logistic regressions in which self-rated health was replaced by psychological problems for which help had been sought (OR 0.81, CI 0.68-0.96), persistent or constantly recurring pain that had lasted for 3 months or more (OR 0.79, CI 0.67-0.94), persistent musculoskeletal pain for at least 3 of the last 12 months (OR 0.79, CI 0.66-0.93), or EQ-5D score (OR 0.78, CI 0.65-0.93). There were no strong correlations (defined as rho >0.5) between the independent variables in any of the models.

**Table 3:** GP users’ probability of CAM provider use*

	**CAM provider use n = 8099**	
	**OR**	**95% ** **CI**
**GP duration**		
0-2 years	1.00	(ref)
>2 years	0.81	0.68-0.96
**GP frequency**		
< 3 visits	1.00	(ref)
3 visits +	1.57	1.37-1.81
**Gender**		
Female	1.00	(ref)
Male	0.45	0.39-0.52
**Age**		
30-49	1.00	(ref)
50-69	0.81	0.70-0.94
70-87	0.56	0.43-0.71
**Marital status**		
Single	1.00	(ref)
Married/cohabitant	0.78	0.65-0.93
Household income**		
Low	1.00	(ref)
Low middle	1.46	1.12-1.90
High middle	1.74	1.29-2.33
High	1.62	1.16-2.27
Education***		
Low	1.00	(ref)
Middle	1.04	0.86-1.25
High	1.16	0.95-1.41
**Self-rated health**		
Poor	1.00	(ref)
Fair	0.62	0.48-0.80
Good	0.45	0.35-0.58
Excellent	0.35	0.26-0.48

CAM, complementary and alternative medical provider; GP, general practitioner; OR, odds ratio; CI, confidence interval.

*Multivariable logistic regression with all left column variables in the model.

**Low (< NOK 200,000), Low middle (NOK 201,000-400,000), High middle (NOK 401,000-700,000), High (> NOK 700,000).

***Low (primary/part of secondary school), Middle (high school), High (college/university).

Other factors associated with higher CAM provider use were more frequent GP visits, female gender, lower age, being single, higher income, and poorer self-rated health, while there was no association with educational level (Table 3). However, the association between CAM use and GP frequency was modified by gender, and the association was stronger in women (interaction term GP frequency x gender, OR 0.72, CI 0.55-0.94). There were no other statistically significant interactions between GP duration or GP frequency and the variables of age, marital status, income, education or self-rated health, either for the whole sample or in separate analyses of genders.

## Discussion

We have shown that the probability of visiting a CAM provider was lower among those with a GP relationship of more than 2 years compared to those with a shorter relationship. The finding remained statistically significant regardless of adjustments with different proxies for health care needs. Women, frequent GP users and GP users in poorer health, lower age and higher income groups had a higher probability of CAM use, whereas there was no difference associated with education.

**Figure 2:**
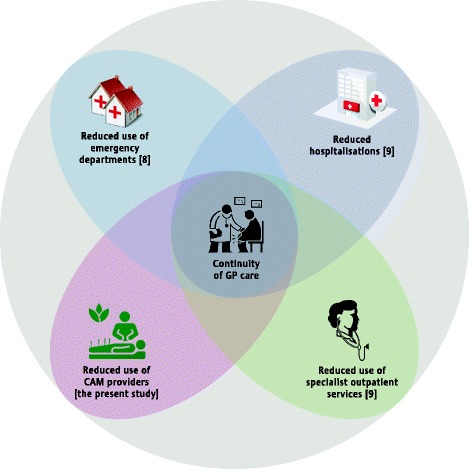
**Reported associations of continuity of general practitioner care and use of health care services.**

The relation between continuity of GP care and use of CAM providers is largely unknown. The present study is among the first to fill this gap. Our main finding adds to findings that continuity of GP care is associated with reduced use of emergency departments, hospitalisation, and outpatient specialist services [9,10] (Figure 2). Because there is little or no gatekeeping for use of emergency departments and CAM providers, continuity itself may contribute significantly to the association, regardless of referrals. Furthermore, continuity may contribute to the association regardless of urgency, since these four health services include emergency as well as elective care. Continuity may thus prevent a leakage of patients from general practice in many different directions, and contribute to a higher degree of treatment and follow-up by the GP, in keeping with the intention of many contemporary health reforms [35-37]. Most patients will find it more satisfactory to receive their necessary care from one provider rather than from many [3-5].

We found that 13.3% of those who had visited a GP during the previous year had also visited a CAM provider. In a previous study of the same Tromsø population where non-GP-users were included, 12.7% had visited a CAM provider and 82% a GP [17]. Similar CAM visit rates and a high GP visit rate suggest that a general population and a GP population may not differ significantly regarding overall CAM use. However, these populations might differ along other parameters, for instance education and income [38]. In the present GP-using sample, CAM providers were more likely to be visited by women and younger individuals (30–49 years) and those in poorer health. This finding is consistent with most studies of general populations [31,39-41]. Contrary to the majority of international research [41], but in concordance with recent Norwegian studies [40,42], we found no association of CAM use with higher education. It is reported that this association weakened from 1985 to 1995 [42]. The educational level in Norway is increasing [43] and Tromsø is above the national average [44]. This may suggest that educational differences levels out as CAM provider use and educational level increases. Regarding income, many international studies report no associations with CAM use [41], whereas we found increased use in higher income groups. However, where significant associations are reported the main direction coincides with our result [41]. One possible explanation may be that CAM provider care is more expensive for the patient than conventional care in Norway, unlike in the USA where most research in this field has been conducted [41].

Frequent GP users were more likely to visit CAM providers than less frequent GP users (Table 3). However, there was an effect modification by gender, as this finding was stronger in women. Women constitute the majority of CAM users, and our finding supports the suggestion that CAM use is additional more than alternative to GP care [21,30,42]. Women assess their health as worse and seek care more often than men [17,45]. Their consultations are longer, include more preventive services, and have a more talkative patient-centred approach, in particular with female doctors [46,47]. Patient empowerment and participation in health care decisions is more likely to be facilitated where patient-centred talk takes place, which increases with consultation time [48]. Frequent GP users with an unsatisfactory GP relationship might use CAM providers more extensively, and/or change their GP. Accordingly, those who hesitate to change their GP may also be those who hesitate to seek health care in general. In a recent study, we found women more likely than men to have a break in continuity of GP care [10]. A patient syndrome of discontinuity has been described [49], and might be part of the explanation for both genders. Another possible explanation is that women might be more sensitive to relational aspects, and have a greater subjective need for an interlocutor in general health and life issues.

Continuity may indicate quality, mutual knowledge and understanding, good communication, and mutual trust in the GP-patient relationship [50]. Conversely, use of CAM providers might indicate distrust and dissatisfaction with the GP and conventional care [18], rather than a belief that conventional care is ineffective [19]. This is consistent with the suggestion that trust and belief in CAM providers is an important reason for CAM use [20], along with other reasons such as seeking to obtain a more holistic view, active participation, and empowerment in care [21]. One might speculate that continuity and CAM use are indications of the same phenomenon, namely the GP’s ability and capacity to deliver on these modalities. GPs’ interest in CAM treatment [51] might be developed by communication about such treatment during the consultations [22,35]. This might ensure that the totality of treatments is beneficial to the patient, and might also strengthen the GP’s coordinating role in health care [23,35]. Primary care physicians often borrow the famous words of Terence (170 BC) “I consider nothing that is human alien to me” [52], and we could add “not even my patients’ use of CAM.”

Particular strengths of this study were the large sample size, the high response rate, and the comprehensive coverage of information about health, disease, and socio-economic status in the questionnaires.

The study had some shortcomings. Despite a high response rate, our sample may not be entirely representative of the general population, as it is well known that women, married people/cohabitants, healthier persons, and higher socio-economic groups are more likely to participate in population surveys [53]. In Tromsø 6, attendees were older, and the proportions of married people/cohabitants and women were higher than for non-attendees [32,33]. In the question “For how long have you had your current GP/other doctor?” some participants might have thought of a specialist physician as their current doctor. Some may have reported visits to other GPs than their current one, for instance due to the doctor’s absence for various reasons. However, a Norwegian study of continuity reported that 78% of consultations were with the usual GP [54], making it unlikely that doctors’ absence has influenced our results significantly. Further, GP duration as a measure of continuity may be a subject of discussion since elements of intensity of care are often included in the continuity term [1,3]. However, because we used a GP visiting sample and because our models were adjusted for GP frequency, the aspect of intensity of care as a part of the continuity term is addressed in our analyses. Besides, the Norwegian list system is considered suited for continuity of care [26-28]. In interpreting our results, one should be aware that there are considerable inconsistencies in the literature regarding characteristics of CAM users [41]. Comparisons should be made with caution due to differences in definitions of CAM and CAM use, study populations, designs, analyses, supply of services, cultures, general living conditions, and health care systems available to the populations studied [41,55]. There is also a potential for recall bias and underreporting, as the use of some CAM providers might not be regarded as socially acceptable. Further, the validity of self-reported data may be questioned, although agreement between self-reported and registered health care use is generally high [56]. Our measures of CAM use have not been validated due to lack of registry data. The same applies to GP duration and GP frequency, where registry data might have been used for validation purposes. Finally, we cannot exclude the possibility of unmeasured confounders of the reported associations, such as GP age, gender, and other GP characteristics. This similarly applies to patient factors such as illness beliefs, coping strategies, and expectations of health care services.

## Conclusions

We concluded that continuity of care, as measured by self-reported duration of the relationship with a named GP, was associated with reduced use of CAM providers. Even if these associations are not proofs of causality, they might add to a pattern from previous studies indicating that continuity of GP care contributes to health care delivery from fewer providers than non-continuity.

## Abbreviations

CAM: Complementary and Alternative Medicine
CI: Confidence Interval
EQ-5D: Euro Quality of Life Group five Dimensions score scale
GP: General Practitioner
NOK: Norwegian Kroner
OR: Odds ratio
RHO: Spearman’s rank correlation
Tromsø 6: The sixth Tromsø Study
